# Socioeconomic deprivation and mode-specific outcomes in patients with chronic heart failure

**DOI:** 10.1136/heartjnl-2017-312539

**Published:** 2018-01-31

**Authors:** Klaus K Witte, Peysh A Patel, Andrew M N Walker, Clyde B Schechter, Michael Drozd, Anshuman Sengupta, Rowenna Byrom, Lorraine C Kearney, Robert J Sapsford, Mark T Kearney, Richard M Cubbon

**Affiliations:** 1 Multidisciplinary Cardiovascular Research Centre, The University of Leeds, Leeds, UK; 2 Department of Family and Social Medicine, Albert Einstein College of Medicine, Bronx, New York, USA; 3 Department of Cardiology, Leeds General Infirmary, Leeds, UK

**Keywords:** heart failure, hospitalisation, deprivation, socioeconomic, mortality

## Abstract

**Objective:**

To characterise the association between socioeconomic deprivation and adverse outcomes in patients with chronic heart failure (CHF).

**Methods:**

We prospectively observed 1802 patients with CHF and left ventricular ejection fraction (LVEF) ≤45%, recruited in four UK hospitals between 2006 and 2014. We assessed the association between deprivation defined by the UK Index of Multiple Deprivation (IMD) and: mode-specific mortality (mean follow-up 4 years); mode-specific hospitalisation; and the cumulative duration of hospitalisation (after 1 year).

**Results:**

A 45-point difference in mean IMD score was noted between patients residing in the least and most deprived quintiles of geographical regions. Deprivation was associated with age, sex and comorbidity, but not CHF symptoms, LVEF or prescribed drug therapy. IMD score was associated with the risk of age-sex adjusted all-cause mortality (6% higher risk per 10-unit increase in IMD score; 95% CI 2% to 10%; P=0.004), and non-cardiovascular mortality (9% higher risk per 10-unit increase in IMD score; 95% CI 3% to 16%; P=0.003), but not cardiovascular mortality. All-cause, but not heart failure-specific, hospitalisation was also more common in the most deprived patients. Overall, patients spent a cumulative 3.3 days in hospital during 1 year of follow-up, with IMD score being associated with the age-sex adjusted cumulative duration of hospitalisations (4% increase in duration per 10-unit increase in IMD score; 95% CI 3% to 6%; P<0.0005).

**Conclusions:**

Socioeconomic deprivation in people with CHF is linked to increased risk of death and hospitalisation due to an excess of non-cardiovascular events.

## Introduction

The prevalence of chronic heart failure (CHF) continues to rise, due to both improving survival from its antecedents, such as ischaemic heart disease, and reducing mortality in patients with established CHF.^1–3^ As such, the personal and economic burdens of the disorder are increasingly relevant to healthcare planning and policy. Hospitalisation is the principal contributor to the healthcare costs of patients with CHF, which itself accounts for almost 2% of the UK National Health Service budget.^3^ Identification of avoidable causes of hospital admission therefore has the potential to define important changes in practice and resource allocation. Socioeconomic status has long been recognised as an important cause of healthcare-related inequalities^4 5^; however, its relevance to the burden of hospitalisation in patients with CHF is unexplored. A recent systematic review has suggested increased risk of hospitalisation in patients with CHF with lower socioeconomic status, as defined by a diverse range of economic, educational and composite markers.^6^ However, these studies recruited participants during hospital admissions, who were managed with now outdated therapies, and so are of unclear relevance. Moreover, no link to the personal and economic burden of hospitalisation was made in these studies, as they did not define the number and duration of hospitalisations. Furthermore, the literature regarding whether socioeconomic deprivation is associated with mortality in patients with CHF is conflicting, and has not addressed the mode of death.^7–9^ To address these important uncertainties, we aimed to define whether mode-specific hospitalisation and mortality are associated with an established composite index of socioeconomic status in a cohort of well-characterised patients with stable CHF and left ventricular (LV) systolic dysfunction.

## Methods

We conducted a prospective cohort study, aiming to examine factors associated with outcome in 1802 unselected patients with CHF treated with ‘state of the art’ therapies, carried out in cardiology outpatient clinics of four UK hospitals between June 2006 and July 2014. All patients provided written informed consent to participate; the investigation complies with the principles of the Declaration of Helsinki. Patients were eligible for inclusion if they were ambulant outpatients with stable clinical signs and symptoms of CHF for 3 months, with an LV ejection fraction of ≤45% defined by transthoracic echocardiography. This LV ejection fraction cut-off was predefined, based on risk of adverse outcomes demonstrating a threshold effect at this level.^1 10^ All consecutive clinic patients meeting inclusion and exclusion criteria were approached to participate.

### Baseline assessment

All patients underwent resting 12-lead ECGs, and blood testing for measurement of full blood count, electrolytes and serum creatinine. Estimated glomerular filtration rate (eGFR) was calculated using the Modification of Diet in Renal Disease method.^11^ Functional status was assessed using the New York Heart Association (NYHA) classification.^1^ Two-dimensional transthoracic echocardiography was performed in all patients and reported by local cardiac sonographers, blinded to patient characteristics, according to British Society of Echocardiography recommendations^1^; LV ejection fraction was calculated according to the Simpson’s biplane method. Doses of diuretic therapy, ACE inhibitors, angiotensin receptor blockers and beta-blockers were collected at recruitment; these were normalised to maximum licensed CHF dose as previously described.^1^ Receipt of cardiac device therapy (either cardiac resynchronisation therapy or implantable cardioverter-defibrillator) was assessed during the 6-month period after recruitment. Data were complete for all variables, other than systolic blood pressure (missing n=284), heart rate (missing n=158), QRS interval (missing n=157), haemoglobin (missing n=20), eGFR (missing n=8), LV ejection fraction (missing n=45) and medication doses (missing n=5); at least one of these variables was missing in 485 cases. In a subset of 408 patients, clinical review was repeated approximately 1 year later to document CHF drug titration, along with changes in symptomatic status, renal function and LV dimensions (remodelling). This subset represents all patients within the first 628 cohort participants who were alive and willing to attend a study follow-up visit, as previously described.^12^

### Socioeconomic status

Individual patient postal codes were used to define allocation to 1 of 32 482 geographical regions, each accounting for approximately 1500 people, for which the Index of Multiple Deprivation (IMD) defines local socioeconomic deprivation.^13^ As our cohort recruitment spanned three official updates to IMD (2007, 2010 and 2015), we allocated IMD rank/score using the update closest to an individual’s recruitment date. IMD provides a contemporaneous index of socioeconomic deprivation, compiled using data collected by multiple UK government and non-government agencies, and is recognised as a valid marker of overall deprivation at a geographical level.^6 9^ Specifically, this provides a composite deprivation score for each region, weighted according to domains of: income (22.5%); employment (22.5%); health and disability (13.5%); education, skills and training (13.5%); barriers to housing and services (9.3%); crime (9.3%); and living environment (9.3%). In some analyses, we have divided these 32 482 regions into quintiles, with 1 denoting the least deprived, and 5 the most deprived. In other analyses, we have used the IMD score within these 32 482 zones allocated to individual patients as a continuous measure of deprivation (across a range of 1.6–78.4 arbitrary units).

### Hospitalisation and mortality

Non-elective hospitalisation was assessed within 1 year after enrolment using recruiting hospital clinical event (Patient Administration System) databases detailing the nature and duration of admissions. Each hospitalisation was subclassified independently by two cardiologists as cardiovascular if the principal presenting complaint was related to cardiac, cerebrovascular or peripheral vascular disease; consensus was sought in all initial cases of disagreement. Heart failure (HF)-related admissions were further subclassified if the patient presented with symptoms and signs of HF and evidence of fluid overload requiring intravenous diuretic therapy for at least 24 hours. All patients were registered with the UK Office of Population Censuses and Surveys to provide details of death until the censoring date of 8 May 2016. Mode of death was subclassified, according to our published methods,^1^ as: (1) sudden cardiac, if it occurred within 1 hour of a change in symptoms or during sleep, or while the patient was unobserved; (2) progressive HF, if death occurred after a documented period of symptomatic or haemodynamic deterioration; (3) other cardiovascular death, if not occurring suddenly or in association with progression of HF; and (4) non-cardiovascular death. For the purpose of this analysis, deaths are simply classified as cardiovascular or non-cardiovascular.

### Statistics

Data are presented as mean (SE of the mean) or percentage (number) for continuous and categorical variables, respectively. Continuous data are compared across IMD quintiles using analysis of variance for normal data, or Kruskal-Wallis tests where indicated as non-normal. Categorical data are compared across IMD quintiles using χ^2^ tests. Zero-inflated Poisson regression analyses applying an offset of total days in study period were used to model the relationship between IMD score and the total number of days a patient was hospitalised, or the total number of hospitalisations, since the distribution of these outcome measures contained a large proportion of zeros. Calibration plots for these models, along with the rationale for their use, are presented in the online supplementary figures 1–3. The analysis that adjusted for all covariates was affected by appreciable amounts of missing data. Consequently, we applied multiple imputation, using a multivariate normal distribution for the continuous variables, and a multinomial logistic imputation for NYHA class (the only discrete variable with missing values). Results from 100 imputed data sets were combined according to Rubin’s rules.^14^ Crude mortality analyses across quintiles were performed using log-rank tests and Kaplan-Meier curves were constructed to illustrate these. Cox regression analysis was then used to model the age-sex adjusted association between IMD score and risk of death, along with further multivariable mortality analyses; global Schoenfeld residual tests and plots of residuals against time confirmed no deviation from proportional hazards assumptions. Competing risk analyses (Fine and Gray’s proportional hazards models with no adjustment for covariates) were used to perform supplementary analyses of cardiovascular and non-cardiovascular death, with data plotted in cumulative incidence function curves. Analyses were conducted using SPSS V.21 (IBM) and Stata V.14.2 (StataCorp). Statistical significance was defined as P<0.05.

10.1136/heartjnl-2017-312539.supp1 Supplementary material 1

## Results

Within the cohort of 1802 patients, 13.8% were in IMD quintile 1 (least deprived), 20.7% in quintile 2, 16.2% in quintile 3, 19.3% in quintile 4 and 30% in quintile 5. Descriptive data comparing the demographics, comorbidity, severity and management of HF in these groups are outlined in table 1. The quintiles appear broadly similar in terms of HF symptoms, LV function and doses of standard HF medications. However, the most deprived quintiles were: younger; less likely to be male; more likely to suffer from chronic obstructive pulmonary disease (COPD); had better renal function; had higher systolic blood pressure and heart rate; had narrower QRS interval; and were less likely to receive cardiac device therapy (which is expected in the context of narrower QRS interval).

**Table 1:** Patient characteristics are associated with socioeconomic status

	1	2	3	4	5	P value
	n=249	n=373	n=292	n=347	n=541	
Male sex (% (n))	77.1 (192)	77.2 (288)	71.2 (208)	74.9 (260)	68.6 (371)	0.017
Age (years)	71.4 (0.7)	71.5 (0.6)	70.9 (0.7)	70.4 (0.7)	66.3 (0.6)	<0.001
NYHA class (% (n))						0.11
1	21.3 (53)	19 (71)	21.3 (62)	15.3 (53)	17.4 (94)	
2	55.8 (139)	51.7 (193)	48.5 (141)	51.3 (178)	48.3 (261)	
3	22.5 (56)	27.9 (104)	29.2 (85)	31.4 (109)	33.3 (180)	
4	0.4 (1)	1.3 (5)	1 (3)	2 (7)	0.9 (5)	
Diabetes (% (n))	22.9 (57)	26.5 (99)	29.1 (85)	26.8 (93)	31.4 (170)	0.13
COPD (% (n))	11.6 (29)	12.3 (46)	14.7 (43)	20.2 (70)	17.7 (96)	0.009
Ischaemic CHF (% (n))	61 (152)	60.1 (224)	57.5 (168)	60.8 (211)	57.7 (312)	0.79
Systolic BP (mm Hg)	118.6 (1.4)	123.4 (1.2)	125 (1.3)	121.3 (1.4)	122.7 (1)	0.022
Heart rate (bpm)	71.9 (1.1)	76.4 (0.9)	73.9 (1.2)	75.2 (1)	77 (0.8)	0.003
QRS width (ms)	124.5 (1.9)	127.5 (1.8)	125.2 (1.9)	124.7 (1.8)	117.6 (1.3)	<0.001
Haemoglobin (g/dL)	13.5 (0.1)	13.5 (0.1)	13.6 (0.1)	13.4 (0.1)	13.4 (0.1)	0.72
eGFR (mL/min/1.73 m^2^)	55.4 (1.1)	56.7 (0.9)	57.7 (1.1)	56.8 (1.1)	60.2 (0.9)	0.006
LV ejection fraction (%)	31.2 (0.6)	31.8 (0.5)	32.6 (0.6)	31.9 (0.5)	32.1 (0.4)	0.54
Ramipril equivalent dose (mg)	5.2 (0.2)	5 (0.2)	5.1 (0.2)	4.5 (0.2)	4.9 (0.2)	0.17
Bisoprolol equivalent dose (mg)	4.2 (0.2)	4.1 (0.2)	3.6 (0.2)	3.7 (0.2)	3.8 (0.1)	0.09
Furosemide equivalent dose (mg)	53.8 (3.2)	50 (2.6)	47.2 (2.5)	53 (2.6)	51.9 (2.3)	0.5
Device therapy (% (n))	36.9 (92)	31.4 (117)	28.4 (83)	28.2 (98)	21.1 (114)	<0.001
IMD score (au)	5.6 (0.1)	11.2 (0.1)	16.9 (0.1)	27.8 (0.2)	51.4 (0.4)	<0.001

au denotes arbitrary units.

BP, blood pressure; CHF, chronic heart failure; COPD, chronic obstructive pulmonary disease; eGFR, estimated glomerular filtration rate; IMD, Index of Multiple Deprivation; LV, left ventricular; NYHA, New York Heart Association.

### Hospitalisation

No statistically significant differences were apparent in the proportion of each quintile hospitalised due to cardiovascular or HF-specific causes during the first year of follow-up (table 2). However, significant differences were noted in the proportion of each quintile with any all-cause hospitalisation, the total number of all-cause hospitalisations per year and the total number of days spent in hospital per year. In order to probe this observation further, zero-inflated Poisson regression analysis was performed to model the age-sex adjusted association between individual patient IMD score and their number of hospitalised days. This suggests that for every 10-unit increase in IMD score (for reference, the IQR in this cohort is 30), the age-sex adjusted number of days hospitalised increased by 4% (95% CI 3% to 6%; P<0.0005). This association was lost when all other variables in table 1 were included in the model using multiple imputation of missing variables (0%; 95% CI −1% to 2%; P=0.51). Importantly, age-sex adjusted zero-inflated Poisson regression analysis also indicated every 10-unit increase in IMD score was associated with a 6% increase in the number of hospitalisations (95% CI 1% to 11%; P=0.009). The magnitude of this association reduced and became statistically non-significant when all other variables in table 1 were included in the model using multiple imputation for missing variables (3% increase; 95% CI −2% to 8%; P=0.2).

**Table 2:** Cause and volume of hospitalisation are associated with socioeconomic status

	IMD quintile					P value
	1	2	3	4	5	
Heart failure hospitalisation (% (n))	6 (15)	5.4 (20)	6.8 (20)	6.9 (24)	6.1 (33)	0.91
Cardiovascular hospitalisation (% (n))	11.2 (28)	10.7 (40)	10.6 (31)	15 (52)	14 (76)	0.24
All-cause hospitalisation (% (n))	24.1 (60)	21.7 (81)	17.8 (52)	33.7 (117)	27.2 (147)	<0.001
Total hospitalisations (events/year)	0.33 (0.04)	0.42 (0.06)	0.33 (0.07)	0.58 (0.06)	0.47 (0.04)	<0.001*
Total hospitalised days (days/year)	3.2 (0.7)	2.7 (0.4)	1.8 (0.3)	4.5 (0.6)	3.9 (0.5)	<0.001*

*Denotes use of Kruskal-Wallis test.

IMD, Index of Multiple Deprivation.

### Mortality

**Figure 1:**
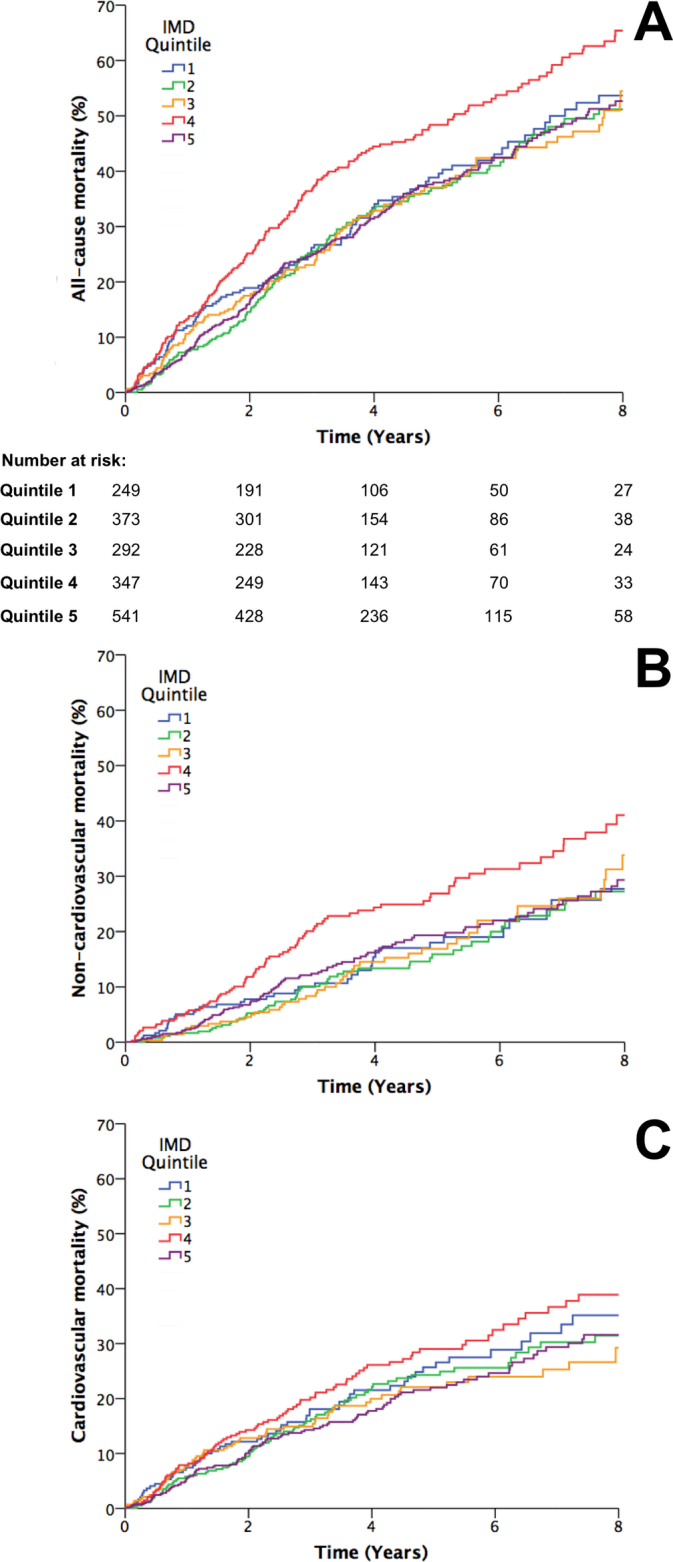
All-cause and mode-specific mortality. (A) Kaplan-Meier curves illustrating all-cause mortality according to Index of Multiple Deprivation (IMD) quintiles (P<0.001 by log-rank test), with numbers at risk indicated below the x-axis. (B) Kaplan-Meier curves illustrating non-cardiovascular mortality according to IMD quintiles (P=0.002 by log-rank test). (C) Kaplan-Meier curves illustrating cardiovascular mortality according to IMD quintiles (P=0.11 by log-rank test).

During a mean 4 years of follow-up, 737 deaths occurred, of which 399 were cardiovascular, 314 were non-cardiovascular and 24 were unclassifiable. Crude all-cause mortality was significantly different between IMD quintiles (figure 1A), with quintile 4 experiencing markedly greater mortality than all other quintiles. Notably, this was mirrored by similar differences in non-cardiovascular mortality (figure 1B), although cardiovascular mortality did not differ across IMD quintiles (figure 1C). Similar conclusions were noted using a competing risks analysis (online supplementary figure 4). Age-sex adjusted Cox regression analyses indicated every 10-unit increase in IMD score was associated with 6% higher risk of all-cause mortality (95% CI 2% to 10%; P=0.004), a 9% higher risk of non-cardiovascular mortality (95% CI 3% to 16%; P=0.003) and a non-significant 3% higher risk of cardiovascular mortality (95% CI −2% to 9%; P=0.21). The association between IMD score and non-cardiovascular mortality was not lost (8%; 95% CI 2% to 15%; P=0.011) even after further accounting for non-cardiovascular comorbidities (diabetes, COPD and eGFR as a marker of renal dysfunction).

### Follow-up clinical data

Using observations from a subcohort planned to undergo follow-up assessment approximately 1 year later (table 3), it is apparent that rates of symptomatic deterioration, a driver of HF-related hospitalisation, did not differ across quintiles. While the change in haemodynamic parameters and renal function was also comparable between groups, there was a suggestion of a more beneficial LV remodelling in the most deprived patients. Dose escalation of CHF drug therapies shown to improve prognosis and symptoms was comparable across quintiles, although the dose of diuretic (thought only to improve symptoms) was reduced less in the most deprived group.

**Table 3:** Association of socioeconomic status with changes in heart failure phenotype and treatment over 1 year

	IMD quintile					P value
	1	2	3	4	5	
	n=60	n=84	n=68	n=83	n=113	
Worsening NYHA class (% (n))	20 (12)	11 (9)	13.2 (9)	18.1 (15)	9.9 (11)	0.27
Change in systolic BP	−3.1 (3.5)	−4.4 (2.6)	6.5 (3.1)	−0.5 (2.7)	−1.2 (2.6)	0.6
Change in heart rate	−2.3 (2.5)	−5.5 (2.3)	−5.9 (2.3)	−0.4 (1.9)	−5.8 (2.2)	0.32
Change in haemoglobin	−0.6 (0.2)	−0.6 (0.1)	−0.5 (0.2)	−0.4 (0.1)	−0.5 (0.2)	0.82
Change in eGFR	−0.2 (1.0)	−4.2 (1.7)	−1.6 (1.0)	−2.9 (1.0)	−0.4 (1.1)	0.12
Percent of baseline LV end-systolic dimension	98.4 (2.5)	92.8 (2.3)	95.3 (1.9)	97.5 (2.1)	90.4 (1.6)	0.028
Change in ramipril equivalent dose	1.3 (0.5)	1.0 (0.4)	1.2 (0.3)	1.0 (0.4)	1.3 (0.4)	0.95
Change in bisoprolol equivalent dose	1.1 (0.5)	1.8 (0.4)	2.0 (0.4)	1.9 (0.3)	2.0 (0.3)	0.6
Change in furosemide equivalent dose	−11.9 (5.9)	−3.7 (4.8)	−2.5 (4.8)	14.6 (5.6)	0.4 (4.8)	0.016

BP, blood pressure; eGFR, estimated glomerular filtration rate; IMD, Index of Multiple Deprivation; LV, left ventricular; NYHA, New York Heart Association.

## Discussion

Our study provides the most comprehensive available assessment of the association between socioeconomic deprivation and adverse outcomes in people with CHF, accompanied by important insights regarding potential underlying factors. While HF symptoms, short-term cardiac remodelling and provision of evidence-based medical therapy were comparable across quintiles of IMD, deprivation was associated with increased all-cause mortality and hospitalisation. Importantly, this risk was explained by increased non-cardiovascular mortality and hospitalisation, suggesting that adverse outcomes associated with deprivation are related to non-cardiovascular factors. Moreover, the increased cumulative duration of hospitalisations in the most deprived patients with CHF has important health economic implications. Overall, our observations provide circumstantial support for a causal link between deprivation and the burden of non-cardiovascular mortality and morbidity in people with CHF, raising the question of whether outcomes can be improved by non-cardiovascular and social interventions.

### Socioeconomic status and hospitalisation

Previous studies have broadly supported the link between socioeconomic deprivation, defined using a diverse range of indices, and rehospitalisation of patients with CHF.^6^ For example, Struthers *et al* have linked deprivation, measured by the Carstairs index (a census-based larger geographic area score than IMD), to the crude and adjusted risk of cardiac readmission in 478 patients with CHF recruited in the UK between 1993 and 1994.^15^ Foraker *et al* showed that living in a low-income area was associated with crude and adjusted all-cause rehospitalisation and mortality, in a large cohort recruited between 1987 and 2004 in the USA.^8^ It is important to emphasise that these studies followed patients with CHF in an era before the routine use of beta-adrenoreceptor antagonists, mineralocorticoid receptor antagonists and device therapy, and so are of unclear relevance to contemporary practice. Furthermore, by recruiting patients during a hospitalisation, the issue of selection bias means that their findings cannot be assumed to apply to all patients with CHF. To our knowledge, no published data describe the link between deprivation and hospitalisation (characterised by its nature, frequency and duration) in an unselected population of patients with CHF. Notably, our work suggests a ‘dose–response’ relationship between deprivation and the cumulative duration of hospitalisation, which may be accounted for by variables included in our multivariate analyses.

### Socioeconomic status and mortality

A recent large UK-based community CHF cohort study consistently showed no link between IMD quintile and risk of death in the years 2000–2007.^9^ Less contemporary data from UK patients with CHF, based on earlier definitions of the IMD score, also concur with these observations.^7^ However, income-based proxies of deprivation have been linked with the risk of hospitalisation or death in patients with CHF, particularly in those with the highest burden of comorbidity.^8^ Our data extend these observations by assessing the association of IMD with mode-specific mortality, allowing us to show that only non-cardiovascular death is linked to IMD-defined deprivation. This is congruent with our wider observations that HF-specific symptoms, hospitalisation and treatment showed no association with IMD, and that cardiac remodelling appeared favourable in the most deprived quintile. Hence, it appears that non-cardiovascular interventions may be required to improve age-sex adjusted mortality in socioeconomically deprived patients with CHF. Further support for this comes from a study of 485 people in Canada with angiographically proven coronary artery disease, in whom socioeconomic deprivation was associated with non-cardiovascular death, but not cardiovascular death.^16^

### Socioeconomic status and provision of evidence-based CHF treatment

In agreement with an earlier large community CHF study in the UK,^9^ we have shown comparable provision of CHF drug therapies known to improve prognosis across IMD quintiles. This may offer some explanation for the comparable cardiovascular mortality and hospitalisation across deprivation groups. Other less contemporary studies have provided conflicting conclusions regarding the equitable prescription of these agents in more deprived groups of patients with CHF.^17 18^ As far as we are aware, our work is the first to address whether the prescribed doses of these agents, and their subsequent titration during follow-up, are comparable across deprivation groups. Again, we found no differences, although device therapy (with defibrillator and/or resynchronisation function) was less frequent in more deprived patients. However, this may appropriately reflect differences in indications for these devices (specifically related to QRS interval), and importantly this was not associated with differing cardiovascular mortality.

### Health economic implications

Although we have not conducted formal health-economic analyses, our data suggest that deprivation is likely to be associated with substantial variation in the costs of caring for people with HF, which accounts for almost 2% of UK healthcare costs.^3^ Across our entire cohort, patients spent a mean 3.3 days hospitalised due to any non-elective cause during 1 year of observation; each day is estimated to cost £400.^19^ We found that a 10-point increase in deprivation is associated with an age-sex adjusted 4% increase in the time spent in a hospital. Placed in the context of a 45-point range in IMD score between quintiles 1 and 5, deprivation per se was associated with 19% more time spent in hospital by patients in the most deprived quintile. Extrapolated across the UK, where approximately 450 000 people have HF with reduced ejection fraction, deprivation may be an important modifiable factor influencing the costs of care.

### Strengths and limitations

It is important to acknowledge the inability of observational studies to define cause-effect relationships, although this design remains the most appropriate for studies of deprivation. While we are able to offer many insights into the wider health and treatment of our cohort, future studies would benefit from assessment of the community health and social support available to patients, along with defining whether delays in access to these might have impacted upon duration or frequency of hospitalisation. Unfortunately, we were only able to describe hospitalisation within the recruiting centres as no robust nationwide reporting system was available when this study began in 2006. This may underestimate hospitalisation across our cohort (or within specific groups), although most patients are admitted to their local (recruiting) hospital. Finally, the use of area-based indices of deprivation, such as IMD, cannot guarantee accurate designation of individual patient deprivation. However, alternative individual patient indices, such as income, may be less useful markers of deprivation in the older patient group we have followed.^20^

Our study has a number of key strengths, including the provision of much more detailed descriptive and outcome data than any prior publication addressing this issue. By defining the frequency and duration of hospitalisations, we provide the first quantification of the personal (and potentially economic) burden of hospitalisation in patients with CHF according to their socioeconomic status. Moreover, we also provide the first assessment of how deprivation is linked with mode-specific mortality in patients with CHF. Furthermore, our assessment of changes in disease status, cardiac remodelling and treatment during follow-up allows us to support our suggestion that non-cardiovascular factors may underpin the adverse outcomes linked to deprivation. Finally, our study of unselected outpatients with stable CHF (as opposed to much of the wider literature pertaining to posthospitalisation cohorts), receiving contemporary therapy, makes our findings of broader relevance to patients attending hospital cardiology clinics.

## Conclusions

We show that individual patient indices of socioeconomic deprivation are associated with all-cause mortality and the cumulative duration of all-cause hospitalisation in patients with CHF receiving contemporary therapy. However, this adverse risk profile is explained by an excess of non-cardiovascular mortality and hospitalisation, and was not associated with inequitable provision of evidence-based CHF treatment. Our data should prompt the design of clinical trials of non-cardiovascular and socioeconomic interventions in order to reduce the personal and economic burden of disease in patients with CHF and low socioeconomic status. It will also be important to define whether our findings are pertinent to patients with other chronic diseases.

**Key messages**

**What is already known on this subject?**

Socioeconomic deprivation is an important cause of healthcare-related inequalities, although conflicting data exist regarding its association with adverse outcomes in people with chronic heart failure.

**What might this study add?**

In spite of comparable provision of evidence-based heart failure therapy, socioeconomic deprivation was associated with an increased risk of all-cause mortality and hospitalisation. However, these were explained by an excess of non-cardiovascular events.

**How might this impact on clinical practice?**

Additional cardiovascular therapies are unlikely to address the adverse outcomes associated with socioeconomic deprivation in people with chronic heart failure; trials of socioeconomic and non-cardiovascular interventions are warranted.
