# Supplementary material for: Socioeconomic deprivation and mode-specific outcomes in patients with chronic heart failure
Source: Heart. 2018 Jan 31;104(12):993–8. doi: 10.1136/heartjnl-2017-312539 (PMC5992368; doi:10.1136/heartjnl-2017-312539)
Supplement: Supplementary material 1 [file heartjnl-2017-312539supp001.pdf]

Supplementary Data

Supplemental Figure 1: Calibration plot for zero-inflated Poisson model of number of hospitalisations per year

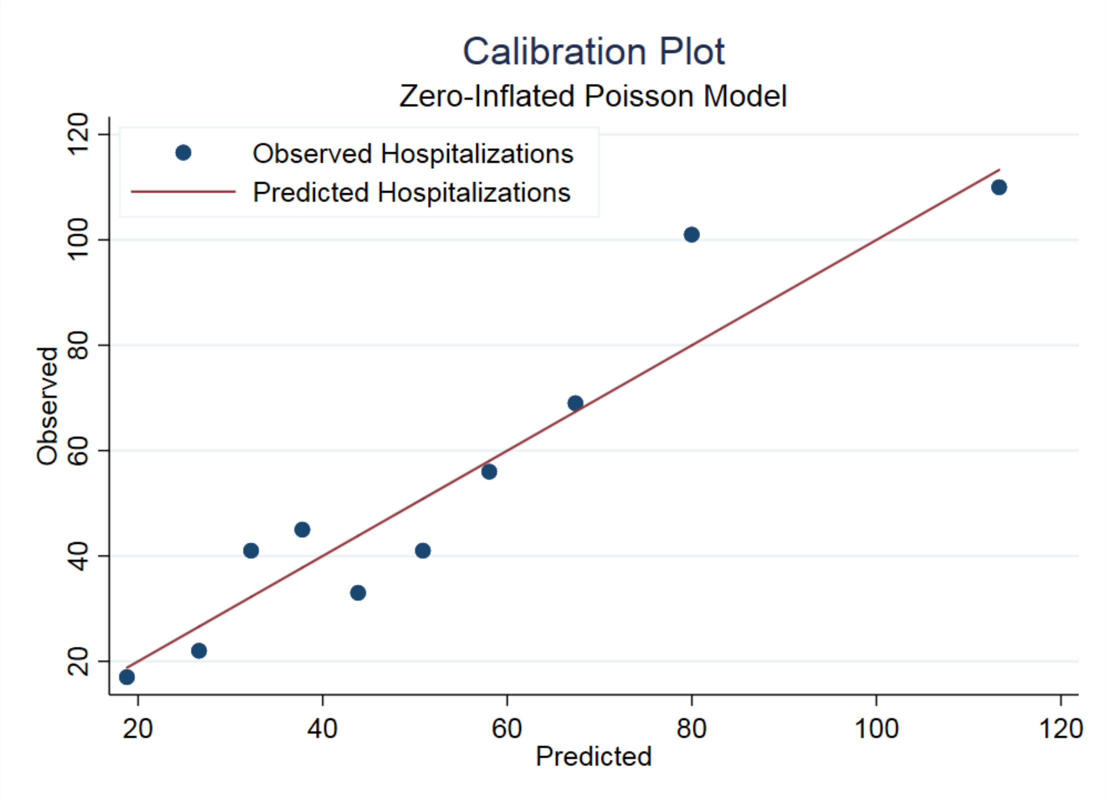

This figure shows the extent of agreement between the observed number of hospitalisations and the number of hospitalisations predicted by the fully-adjusted zero-inflated Poisson model. Each filled circle represents the count of observed (vertical) and predicted (horizontal) hospitalizations in each decile of predicted number of hospitalizations. The diagonal red line is where all points would fall in the event of perfect agreement between the model and observation.

Supplemental Figure 2: Calibration plot for zero-inflated Poisson model of number of hospitalised days per year

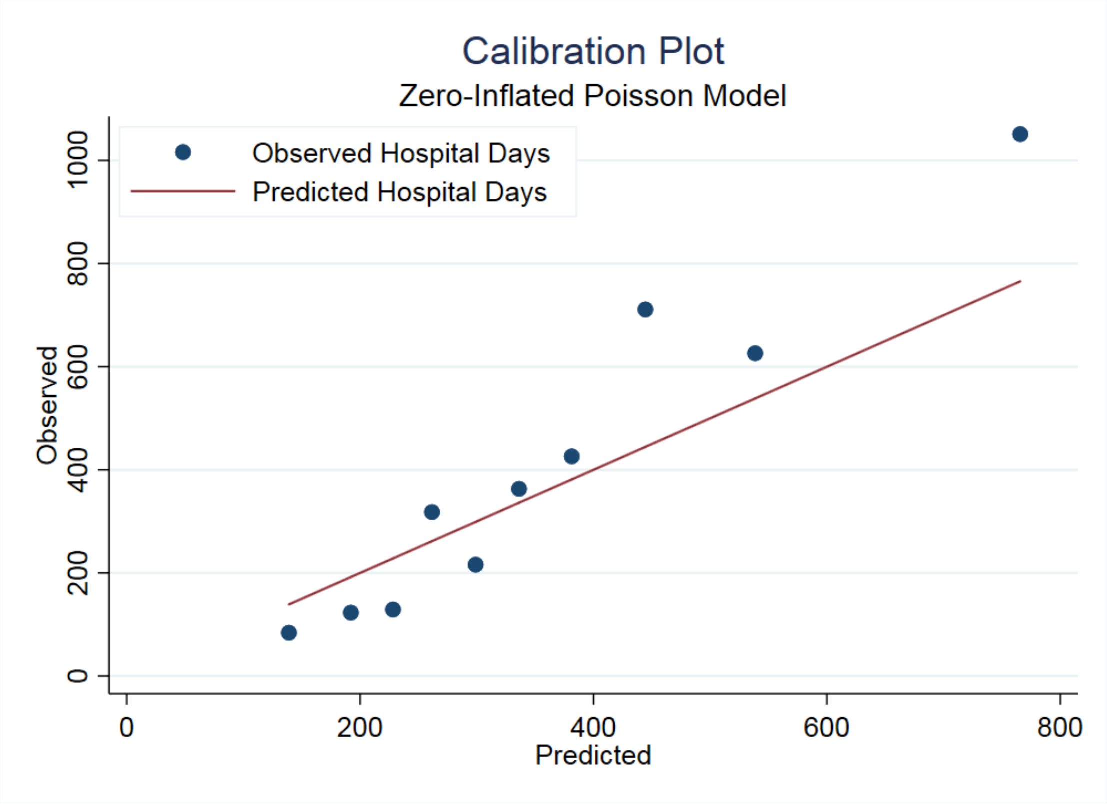

This figure shows the extent of agreement between the observed number of hospitalised days and the number of hospitalised days predicted by the fully-adjusted zero-inflated Poisson model. Each filled circle represents the count of observed (vertical) and predicted (horizontal) hospital days in each decile of predicted number of hospitalised days. The diagonal red line is where all points would fall in the event of perfect agreement between the model and observation.

Supplemental Figure 3: Justification for not using alternate statistical models of the number of hospitalised days per year

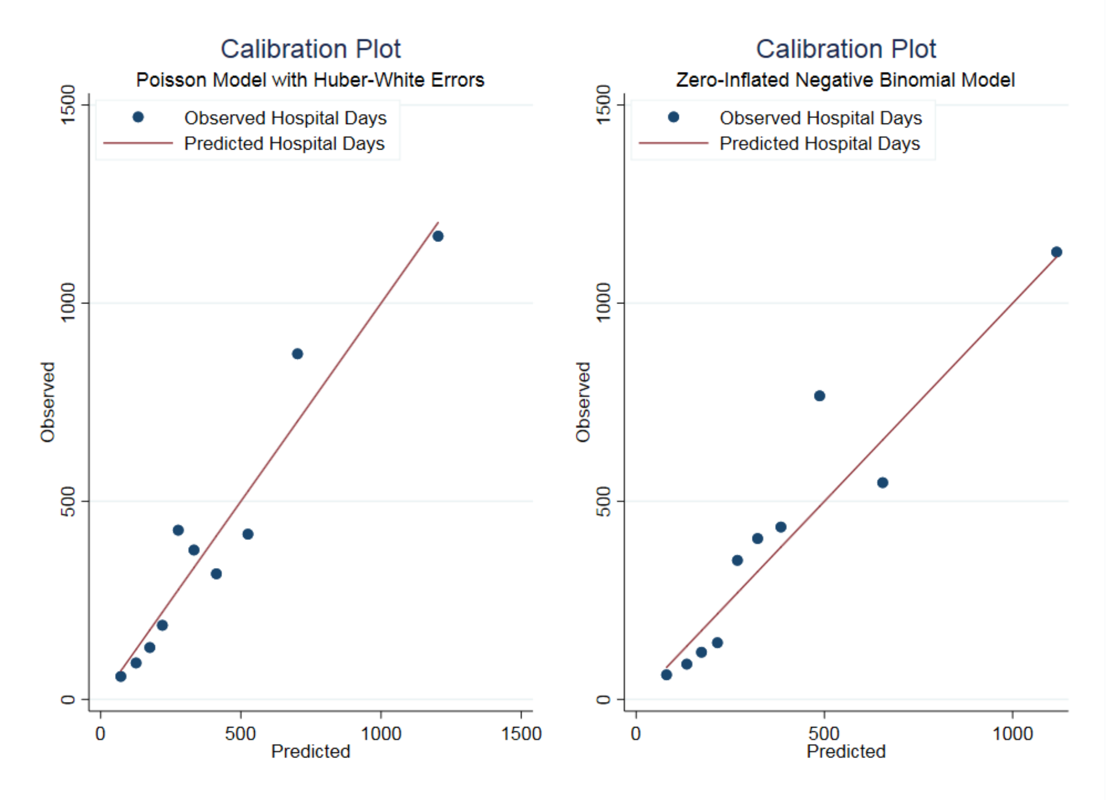

These figures show the extent of agreement between the observed number of hospitalised days and the number of hospitalised days predicted by a fully-adjusted: 1) Poisson model with Huber-White robust standard errors (left); or, 2) zero-inflated negative binomial model (right) to accommodate potential overdispersion. Each filled circle represents the count of observed (vertical) and predicted (horizontal) hospital days in each decile of predicted number of hospital days. The diagonal red line is where all points would fall in the event of perfect agreement between the model and observation. The Poisson model indicates that for every 10-point increase in IMD score, the incidence rate ratio for hospitalized days is 1.12 (95% confidence interval 1.04-1.22), with a Pearson chi square statistic for model fit of 2,990.7, and $p<0.0005$ indicating more than chance disagreement between the data and the analytic model; importantly, confidence intervals for this type of model should be interpreted with caution. The zero-inflated negative binomial model indicates that for every 10-point increase in IMD score, the incidence rate ratio for hospitalized days is 1.05 (95% confidence interval 0.95-1.16) with a Pearson chi square statistic for model fit of 4593.8, and $p<0.0005$ indicating even greater disagreement between the data and the analytic model. The zero-inflated Poisson model presented in supplemental figure 2 is at least as well calibrated as these models (Pearson chi square statistic of model fit of 2886.9 with $p<0.0005$, again indicating more than chance disagreement between the data and the analytic model), and also provides a similar point estimate for the incidence rate ratio. There is no other ideal parametric model to analyse such data.

Supplemental Figure 4: Competing risks analyses

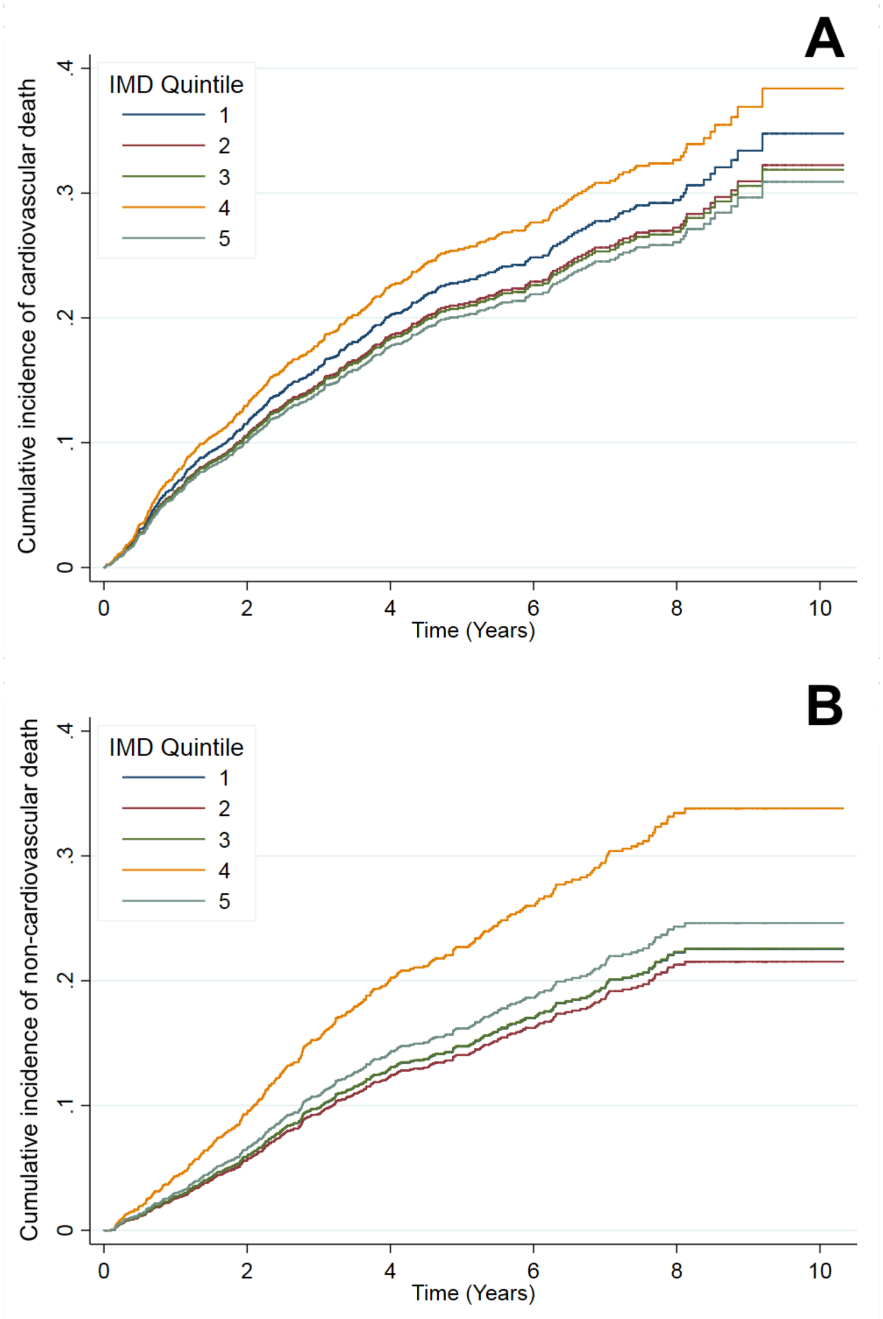

A) Cumulative incidence function illustrating cardiovascular mortality according to IMD quintiles, with non-cardiovascular and unclassifiable mortality as a competing risk (p=0.35 between groups). **B**) Cumulative incidence function illustrating non-cardiovascular mortality according to IMD quintiles, with cardiovascular and unclassifiable mortality as a competing risk (p=0.01 between groups).
